# CircRNA circ_0004370 promotes cell proliferation, migration, and invasion and inhibits cell apoptosis of esophageal cancer via miR-1301-3p/COL1A1 axis

**DOI:** 10.1515/med-2021-0001

**Published:** 2021-01-04

**Authors:** Xiaobo Chen, Hongwen Sun, Yunping Zhao, Jing Zhang, Guosheng Xiong, Yue Cui, Changcheng Lei

**Affiliations:** Department of Thoracic Surgery, The First Affiliated Hospital of Kunming Medical University, No 295 Xichang Road, Kunming 650032, Yunnan, China

**Keywords:** esophageal cancer, circ_0004370, miR-1301-3p, COL1A1

## Abstract

**Background:**

The aim of this study was to investigate the circ_0004370 expression in EC, its effects on cell proliferation, apoptosis, migration, invasion, and epithelial–mesenchymal transition (EMT) process, and the underlying regulatory mechanisms in EC.

**Methods:**

The protein levels of COL1A1 and EMT-related proteins were detected by western blot. The role of circ_0004370 on cell viability, proliferation, and apoptosis was analyzed by Cell Counting Kit-8 (CCK-8) assay, colony formation assay, and flow cytometry, respectively. The transwell assay was used to examine cell migration and invasion. The binding sites between miR-1301-3p and circ_0004370 or COL1A1 were predicted by starbase software and confirmed by dual-luciferase reporter assay and RNA pull-down assay.

**Results:**

We discovered that circ_0004370 was remarkably upregulated in EC tissues and cells. Knockdown of circ_0004370 inhibited cell proliferation, migration as well as invasion, and promoted apoptosis *in vitro*, while its effect was rescued by miR-1301-3p inhibition. And circ_0004370 mediated the EMT process in EC cells. Moreover, we explored its regulatory mechanism and found that circ_0004370 directly bound to miR-1301-3p and COL1A1 was verified as a target of miR-1301-3p. COL1A1 was highly expressed in EC cells and upregulation of COL1A1 reversed the effects of miR-1301-3p on cell proliferation, migration, invasion, and apoptosis. In addition, silencing of circ_0004370 reduced tumor volumes and weights *in vivo*. We showed that circ_0004370/miR-1301-3p/COL1A1 axis played the critical role in EC to regulate the cell activities.

**Conclusion:**

Circ_0004370 promotes EC proliferation, migration and invasion, and EMT process and suppresses apoptosis by regulating the miR-1301-3p/COL1A1 axis, indicating that circ_0004370 may be used as a potential therapeutic target for EC.

## 1 Introduction

Esophageal cancer (EC) is the seventh most common diagnosed cancer and has the sixth highest mortality rate among cancer diseases [1]. There are two subtypes of this disease: esophageal squamous-cell carcinoma (ESCC) and esophageal adenocarcinoma (EAC) [2]. Although a few effective methods such like surgery, chemotherapy, and radiotherapy are used for EC treatment, the survival rate of advanced patients is still less than 20% [3]. Therefore, further exploring the underlying molecular mechanism of EC pathogenesis is quite urgent for researchers to develop novel therapeutic targets for EC patients.

Noncoding RNAs are the type of RNAs that cannot be translated into protein. There are many functional noncoding RNAs such as long noncoding RNAs (lncRNAs), microRNAs (miRNAs), and circular RNAs (circRNAs) [4]. Recently, emerging evidence proved that circRNA might play a critical role in cell biology. CircRNA is formed from the continuous closed-loop structure which means the structure is more stable [5]. Due to its special structure, CircRNA is not easy to degrade compared with normal RNA under the treatment of Actinomycin D or RNase R [6]. In addition, recent publication showed that the circRNA might function as a sponge of miRNA to inhibit the function of miRNA and affect the miRNA target gene [7].

Nowadays, the role of circRNA in EC remains to be elucidated. Research reported that hsa_circ_0004370 expression in EC was dramatically increased than adjacent normal tissues [8]. Nevertheless, the regulatory mechanism and specific function of circ_0004370 in EC cells are not fully clear.

miRNAs are the short noncoding RNA of about 18-24nt. MiRNAs play pivotal roles in gene regulation by binding to mRNAs of targeted protein-coding genes, then inhibits or promotes gene expression in translational level and posttranscriptional level [9]. Thus, miRNA is the key factor for many biological procedures. Many existing studies confirmed that miRNA aberrant expression happened in various diseases [10]. It has been shown that miRNAs are related to the human cancer development and act as tumor promoters or suppressors. The first research found that miR-1301-3p directly bound to oncogene neuroblastoma Ras viral homolog (N-Ras) and acted as a tumor inhibitor in glioma [11]. Besides, miR-1301-3p was suggested to be an effective biomarker for colorectal cancer [12]. Furthermore, a recent article showed that miR-1301-3p repressed cell viability of human breast cancer by directly targeting the immature colon carcinoma transcript 1 (ICT1) [13]. Zhang et al. proved that miR-1301-3p/INCENP axis played the crucial role in the development of ESCC [14], which provided us new insights into the mechanism of miR-1301-3p in EC.

Collagen type I alpha 1 (COL1A1) is a type of collagen. Researchers have discovered that collagen is the important protein component in teeth, bones, the adhesion of tumor cell, and extracellular matrix (ECM) [15,16,17]. In previous studies, COL1A1 was upregulated in cervical cancer cells and restrained cell apoptosis [18]. In addition, a recent study demonstrated that COL1A1 participated in epithelial-to-mesenchymal transition (EMT) process in breast cancer [19]. Furthermore, Yin et al. manifested that COL1A1 enhanced cell proliferation, migration, and invasion in ESCC [20], which implied that COL1A1 was a crucial factor in cancer development and progression.

In this study, we uncovered that knockdown of circ_0004370 in EC was linked to restrain EC cell viability, proliferation, apoptosis, migration, invasion, and *in vivo* tumor formation. In the study of its regulatory mechanism, we found that circ_0004370 bound to miR-1301-3p and inhibited its expression in EC cells. In addition, miR-1301-3p directly targeted COL1A1 and miR-1301-3p overexpression reduced the expression of COL1A1. Thus, a novel regulatory mechanism of circ_0004370/miR-1301-3p/COL1A1 axis could be potential targets for EC treatment and diagnosis.

## 2 Methods and materials

### 2.1 Patients and specimens

Fifty pairs of EC tissues and nearby healthy esophageal tissues were obtained from EC patients diagnosed at the First Affiliated Hospital of Kunming Medical University from April 2018 to January 2019. Detailed clinicopathological features of all patients are shown in the Table 1. All patients wrote informed consents and had not undergone any other treatment. This experiment received the approval from the human ethics committee of the First Affiliated Hospital of Kunming Medical University.

**Table 1:** The correlation between circ_0004370 expression and clinicopathological features of patients with ESCC

Parameters	Low-circ_0004370 (*n* = 25)	High-circ_0004370 (*n* = 25)	*P* value
**Gender**			
Male	16	12	0.254
Female	9	13	
**Age (years)**			
≤60	16	13	0.390
>60	9	12	
**Smoking status**			
Yes	13	7	0.083
No	12	18	
**Histological grade**			
Low or undiffer	11	17	0.087
Middle or high	14	8	
**TNM stages**			
I and II	8	19	0.002**
III	17	6	
**Size**			
≤4 cm	10	18	0.023*
>4 cm	15	7	

*P < 0.05, **P < 0.01.

### 2.2 Cell culture and transfection

The human esophageal adenocarcinoma cell line (OE19) and esophageal squamous-cell carcinoma cell line (KYSE410, EC109 and TE11) were bought from European Collection of Authenticated Cell Cultures (ECACC, Salisbury, UK). Esophageal epithelial cell line of human (HEEC) was obtained from ScienCell Company (San Diego, CA, USA). For the cell culture, all cells were cultured in basal DMEM (Weike Biotechnology, Shanghai, China) containing 10% fetal bovine serum (FBS) at 37°C. OE19 and EC109 cells were used for transfection due to their highest circ_0004370 expression level. MiR-1301-3p mimic and inhibitor, small interfering RNA against circ_0004370 (si-circ #1, si-circ #2 and si-circ #3), their control (miR-NC, anti-NC, and si-NC), and the transfection plasmid vectors pcDNA and COL1A1 were bought from GenePharma Company (Shanghai, China). The procedure of cell transfection obeyed the instructions of Lipofectamine 3000 (Invitrogen, USA). Successful transfected cells prepared in advance were used in the following experiments; si-circ #1 sequence was 5′-GCGUCUCCGUACAGAUGACCATT-3′, si-circ #2 sequence was 5′-GCAGCGAAGGAATAGGACA-3′, si-circ #3 sequence was 5′-GAAGGAATAGGACAACCTT-3′, si-NC sequence was 5′-UUCUCCGAACGUGUCACGUTT-3′.

### 2.3 Actinomycin D assay

To measure the stability of RNA, cells were treated with 2 mg/mL of Actinomycin D (Sigma-Aldrich, St. Louis, MO) for 0, 6, 12, 18, and 24 h. After treated with Actinomycin D, the circ_0004370 and PRRX1 mRNA levels were respectively detected by RT-qPCR assay.

### 2.4 RNA isolation and quantitative real-time reverse transcription-PCR (RT-qPCR)

TRIzol reagent (Invitrogen, Carlsbad, CA, USA) was used to extract total RNA on the basis of the user guide and reverse-transcribed into cDNAs used Transcriptor First Strand cDNA Synthesis Kit (Roche, Indianapolis, IN). RT-qPCR was performed in a 384-well plate containing synthesized cDNA. The results of the expression were presented using 2^−ΔΔCt^ method. GAPDH and U6 acted as controls. We designed the primers for circ_0004370 (forward: 5′-ACCCACCGATTATCTCTCCTG-3′; reverse: 5′-TCCTATTCCTTCGCTGCTTTC-3′), PRRX1 mRNA (forward: 5′-ACGCTTCCCTCCTCAAATCC-3′; reverse: 5′-AGTAGCCATGGCGCTGTACG-3′), miR-1301-3p (forward: 5′-GCCCGCTTGCAGCTGCCTGGGAG-3′; reverse: 5′-GTGCAGGGTCCGAGGT-3′), COL1A1 (forward: 5′-CGATGGATTCCAGTTCGAGT-3′; reverse: 5′-TTTTGAGGGGTTCAGTTTG-3′), U6 (forward: 5′-CTCGCTTCGGCAGCACATATACT-3′; reverse: 5′-ACGCTTCACGAATTT-GCGTGTC-3′), GAPDH (forward: 5′-TGTTCGTCATGGGTGTGAAC-3′; reverse: 5′-ATGGCATGGACTGTGGTCAT-3′).

### 2.5 Localization of nucleus and cytoplasm

In order to study the location of circ_0004370 in EC cell lines, we used the NE-PER™ Nuclear and Cytoplasmic Extraction Reagents Kit (Thermo Scientific). With the instructions on the manufacturer, the EC cells cytoplasm and nuclear components were separated and collected. RT-qPCR was utilized to examine circ_0004370 expression in cell cytoplasm and nucleus. GAPDH is cytoplasm positioning control; U6 is the nucleus positioning control.

### 2.6 Western blotting assay

The RIPA lysis and extraction buffer were the protein extraction buffer used in EC cells. The concentrations of protein were measured with BCA Protein Assay Kit (Beyotime, Shanghai, China). Proteins were separated by sodium dodecyl sulfate polyacrylamide gel electrophoresis (SDS-PAGE). After 120 minutes, the proteins were transferred to the polyvinylidene fluoride (PVDF) membrane and blocked with 5% milk. Primary antibodies anti-GAPDH (1:1,000; Cell Signaling Technology, Danvers, MA, USA), anti-COL1A1 (1:1,000; Abcam, Cambridge, United Kingdom), anti-E-cadherin (1:1,000; Abcam), anti-N-cadherin (1:1,000; Abcam), and anti-Vimentin (1:1,000; Abcam) seeded into membrane at 4°C overnight. And then the complexes were incubated with secondary antibodies (HRP-conjugated, 1:1,000; Abcam). Finally, the ECL method (Thermo Scientific, Waltham, MA, USA) was used for observation and detection.

### 2.7 Cell viability assay

To analyze the cell viability, we used Cell Counting Kit-8 (Beyotime, Shanghai, China) assay. The cells need to be incubated into 96-well plates and then added with CCK-8 solution. The optical density (OD) values were detected at 450 nm in 0, 24, 48, and 72 h.

### 2.8 Cloning formation assay

More than 200 cells were added to the 6-well plate, and the medium contained 10% FBS. First, the cells were fixed with 4% paraformaldehyde (PFA) and stained with crystal violet after two weeks. The number of colonies was counted when the diameters of visible colonies were greater than 1 mm.

### 2.9 Cell apoptosis assay

The cells were first washed with PBS and then resuspended in binding buffer. FITC Annexin V/propidium iodide (PI) (BD Pharmingen, San Diego, CA, USA) was used to double stain the cells according to the manufacturer’s manual. The cells were detected by the flow cytometer (Cytek Biosciences, model: DxP 10).

### 2.10 Transwell migration and transwell invasion assay

Cell migration was determined by transwell. First, cells were cultured in serum-free medium for 24 h. Then 100 µL cells washed with sterile PBS were added to the upper chamber. 600 µL DMEM containing FBS was added to the lower chamber. After cultured for 24 h, the cells were washed twice with sterile PBS and stained with 0.1% crystal violet. The number of cells represented the ability of cell migration. The matrigel (CorningLife Sciences, Corning, NY, USA) was added to the upper chamber in invasion experiment, and all other steps were similar with transwell migration assay. We observed the migration and invasion of cells using a 100× microscopic field.

### 2.11 Dual-luciferase reporter assay

The binding sites between circ_0004370 and miR-1301-3p and miR-1301-3p and COL1A1 were predicted by online software. The sequences of wild type circ_0004370 (WT-circ_0004370), mutant circ_0004370 (MUT-circ_0004370), wild type COL1A1 3′UTR, and mutant COL1A1 3′UTR involving the putative-binding sites of miR-1301-3p were cloned into the pMIR-REPORT luciferase vector (OBio Biology, Shanghai, China). The firefly and rellina luciferase reporter plasmids and miR-1301-3p or miR-NC were cotransfected into EC cells using Lipofectamine 3000 (Invitrogen). The correlation results were detected by the dual-Luciferase reporter system.

### 2.12 RNA pull-down assay

RNA pull-down assay was utilized to detect potential target relationship between circ_0004370 and miR-1301-3p. The biotinylated miR-1301-3p (bio-miR-1301-3p) was purchased from GenScript Biotech Co., Ltd. (Nanjing, Jiangsu, China). 1 × 10^6^ EC109 or OE19 cells were seeded into 6-well plates and then treated with Bio-miR-1301-3p or Bio-NC at a final concentration of 50 nM. After 48 h, EC cells were harvested and lysed. The streptavidin-coupled Dynabeads (Invitrogen, Carlsbad, CA, USA) was used to pull down the biotin–miRNA–circRNA complexes in cell lysates. After washing, the RNA complexes bound to the beads were extracted and subjected to RT-qPCR assay.

### 2.13 Xenograft mouse model

Animal experiments were followed by National Institutes of Health guidelines. The female nude mice (4-week-old) with no specific pathogen-free (SPF) were subcutaneously injected with EC cells transfected with sh-NC or sh-circ_0004370 at a concentration of 5 × 10^6^ cells/200 µL in sterile saline. To analyze the tumor size, the tumor volumes were detected every 7 days and tumor weights were determined after the animals were sacrificed. All the animal studies were approved by the Animal Care committee of the First Affiliated Hospital of Kunming Medical University.

### 2.14 Statistical analysis

GraphPad Prism 7.0 and SPSS 20.0 software were used to fulfil a statistical analysis. The difference between two groups was analyzed by the Student’s *t*-test. In addition, the difference among multiple groups was detected by one-way Analysis of Variance (ANOVA). Data were showed as mean ± standard deviation. *P* value < 0.5 was considered significant.

## 3 Results

### 3.1 The expression level of circ_0004370 was increased in EC

**Figure 1:**
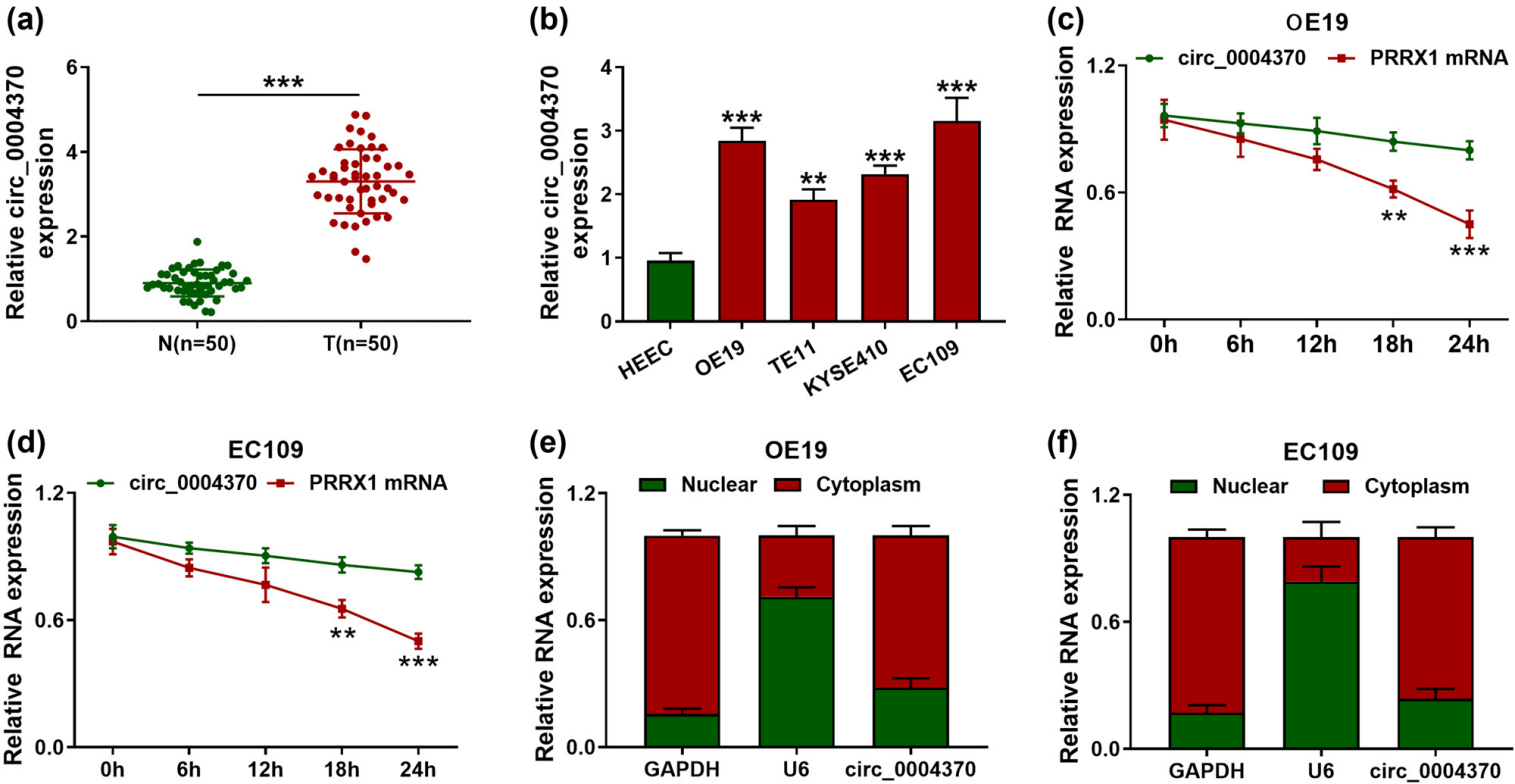
The expression levels of circ_0004370 in EC tissues and cells. (a) Relative expression of circ_0004370 in EC tissues and adjacent normal tissues was detected by RT-qPCR, *n* = 50. (b) Relative expression of circ_0004370 in OE19, TE11, KYSE410, and EC109 and normal cell HEEC was detected by RT-qPCR. (c) Relative RNA expression of circ_0004370 and PRRX1 mRNA in OE19 cells was detected by RT-qPCR. (d) Relative RNA expression of circ_0004370 and PRRX1 mRNA in EC109 cells was detected by RT-qPCR in different times after treatment of Actinomycin D. (e) Relative RNA expression of circ_0004370 in OE19 cell nucleus and cytoplasm was detected by RT-qPCR. (f) Circ_0004370 distribution in EC109 cells was detected by RT-qPCR. **P < 0.01, ***P < 0.001.

In order to know the function of circ_0004370 in EC, the RT-qPCR was used to analyze the expression of circ_0004370 in EC tissues and cells. The expression level of circ_0004370 in EC tissues was significantly higher than that in the adjacent normal controls (Figure 1a). There was the correlation between circ_0004370 expression and clinicopathological features of ESCC patients (Table 1). The expression of circ_0004370 was increased in OE19, TE11, KYSE410, and EC109 cells compared with the HEEC cells, and the increase of expression was most obvious in OE19 and EC109 cells, so these two cell lines were selected for future experiments (Figure 1b). Furthermore, the circ_0004370 circular structure was more stable than the linear structure of the PRRX1 mRNA in OE19 and EC109 cells (Figure 1c and d). We found that circ_0004370 mainly existed in the cytoplasm in OE19 and EC109 cells (Figure 1e and f). These data suggested that circ_0004370 might play a vital role in EC progression.

### 3.2 Circ_0004370 knockdown suppressed EC cell proliferation, migration, and invasion and induced cell apoptosis

**Figure 2:**
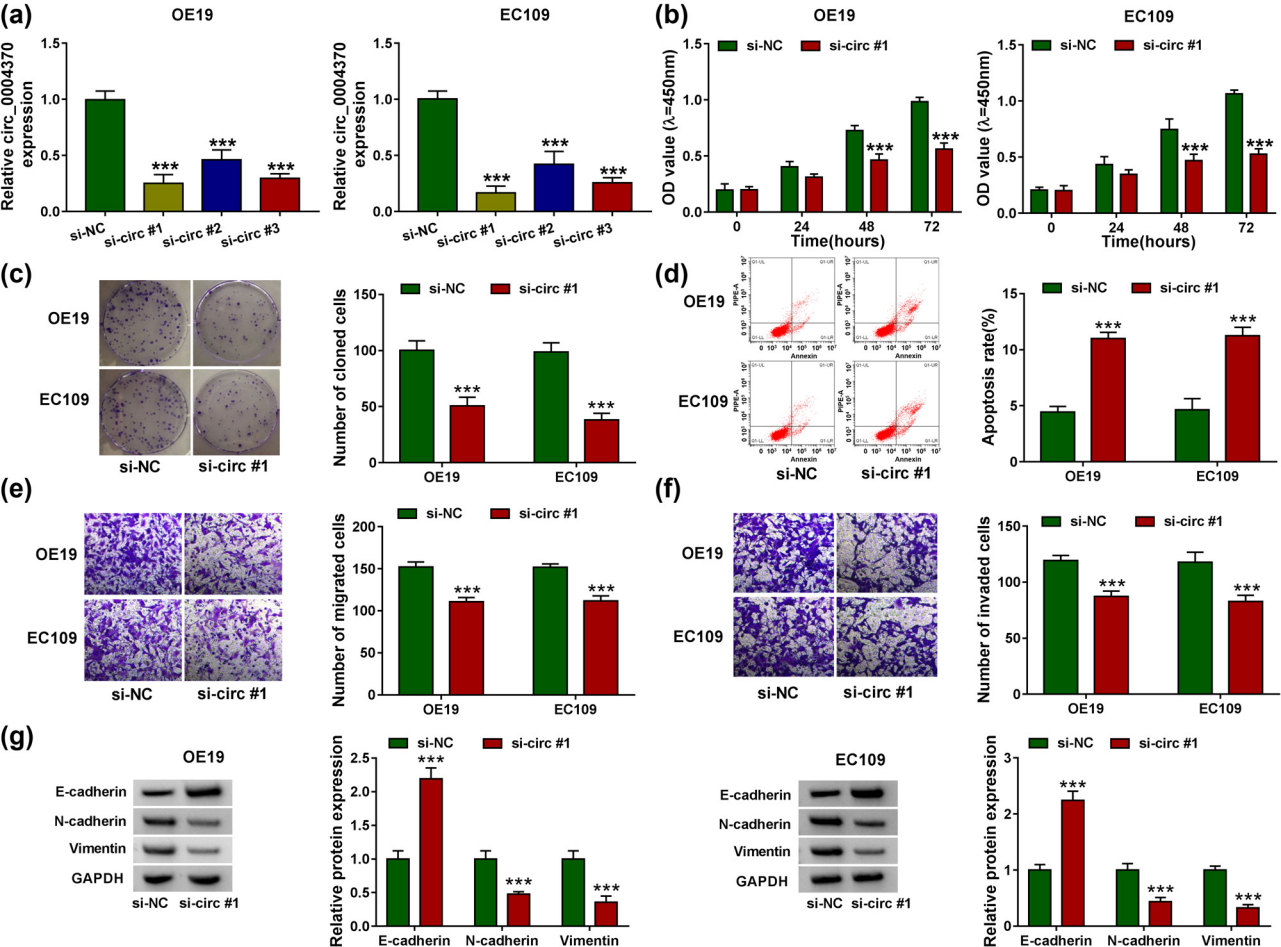
Circ_0004370 regulated EC progression *in vitro*. (a)The relative expression of circ_0004370 in cells was measured by RT-qPCR after transfected with si-NC, si-circ #1, si-circ #2, and si_circ #3 individually. (b) Cell proliferation of OE19 and EC109 cell lines was evaluated by CCK-8 assay. (c) Cell cloning of OE19 and EC109 cell lines was evaluated by Colony formation assay. (d) Cell apoptosis of OE19 and EC109 cell lines was evaluated by flow cytometry assay. (e and f) Cell migration and invasion of OE19 and EC109 cell lines were analyzed by transwell assay. (g) Relative Protein expression of the EMT marker E-cadherin, N-cadherin, and Vimentin was evaluated by western blot analysis in OE19 and EC109 cells. ***P < 0.001.

Loss-of-function experiments were performed to observe whether circ_0004370 affected the behavior of EC cells. After transfection with si-NC, si-circ #1, si-circ #2, or si-circ #3 in EC cells, it was found that the expression level of circ_0004370 was significantly decreased in OE19 and EC109 cells, and because of the better efficiency of si-circ #1, it was used in the subsequent experiments (Figure 2a). CCK-8 assay determined that knocking down circ_0004370 significantly decreased OE19 and EC109 cell viability (Figure 2b). In addition, colony formation assay revealed that downregulation of circ_0004370 significantly inhibited cell proliferation (Figure 2c). Furthermore, flow cytometry indicated that the cell apoptosis in circ_0004370 knockdown group was markedly increased in OE19 and EC109 cells (Figure 2d). Then, the results of transwell assay indicated that cell migration and invasion were reduced in the transfecting with si-circ #1 group in OE19 and EC109 cells (Figure 2e and f). Moreover, the EMT-related proteins were analyzed by western blot assay. Western blot analysis indicated that the protein level of E-cadherin was markedly upregulated after knocking down circ_0004370 in OE19 and EC109 cells, whereas the protein levels of N-cadherin and Vimentin were downregulated after knocking down circ_0004370 in OE19 and EC109 cells (Figure 2g). Thus, these results confirmed that downregulated circ_0004370 suppressed biological activities and EMT process in EC cells.

### 3.3 MiR-1301-3p was a direct target of circ_0004370

**Figure 3:**
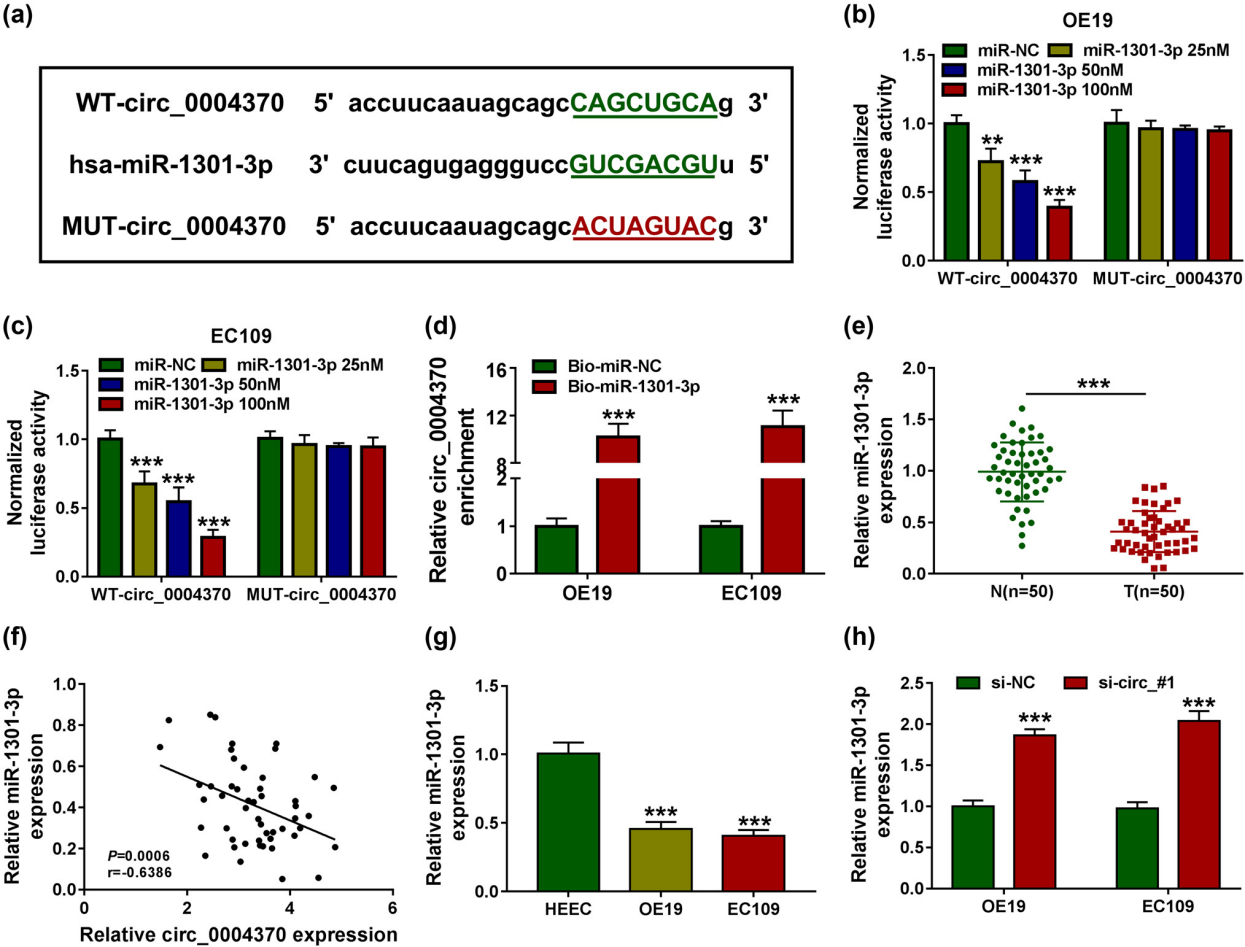
MiR-1301-3p was a direct target of circ_0004370. (a)The binding site of circ_0004370 and miR-145-5p was measured by starBase v2.0. (b–d) The interaction between miR-1301-3p and circ_0004370 in OE19 and EC109 cells was determined by dual-luciferase reporter assay and RNA pull-down assays in OE19 and EC109 cells. (e) Relative miR-1301-3p expression in EC tissues and in adjacent normal tissues was measured by RT-qPCR. (f) The correlation between miR-1301-3p and circ_0004370 levels was measured. (g) Relative miR-1301-3p expression in OE19, EC109, and HEEC cells was detected by RT-qPCR. (h) After transfecting si-NC or si-circ_#1, the expression level of miR-1301-3p was detected by RT-qPCR. ***P < 0.001.

Circ_0004370 was predicted to contain the binding sites with miR-1301-3p using starBase v2.0 software (Figure 3a). To further understand the relationship between circ_0004370 and miR-1301-3p, the dual-luciferase reporter assay was performed. After overexpression of miR-1301-3p with different concentrations of miR-1301-3p mimics (25, 50, and 100 nM, respectively), the luciferase activity in OE19 and EC109 cells containing the WT-circ_0004370 was decreased in a dose-dependent manner, while the luciferase activity in MUT-circ0004370 group was not changed in OE19 and EC109 cell lines (Figure 3b and c). Then the RNA pull-down assay was utilized to further verify the correlation between circ_0004370 and miR-1301-3p. The results presented that circ_0004370 was more enriched in bio-miR-1301-3p-transfected EC cells when compared with bio-NC-transfected EC cells (Figure 3d). Moreover, miR-1301-3p expression level was significantly reduced in EC tissues and cells (Figure 3e and g). The expression of miR-1301-3p had a negative correlation with the expression of circ_0004370 in EC tissues (Figure 3f). Also, miR-1301-3p was upregulated by circ_0004370 knockdown in OE19 and EC109 cells (Figure 3h). From these data, it was indicated that miR-1301-3p was a direct target of circ_0004370.

### 3.4 MiR-1301-3p inhibitor partially rescued the functions of circ_0004370 knockdown

**Figure 4:**
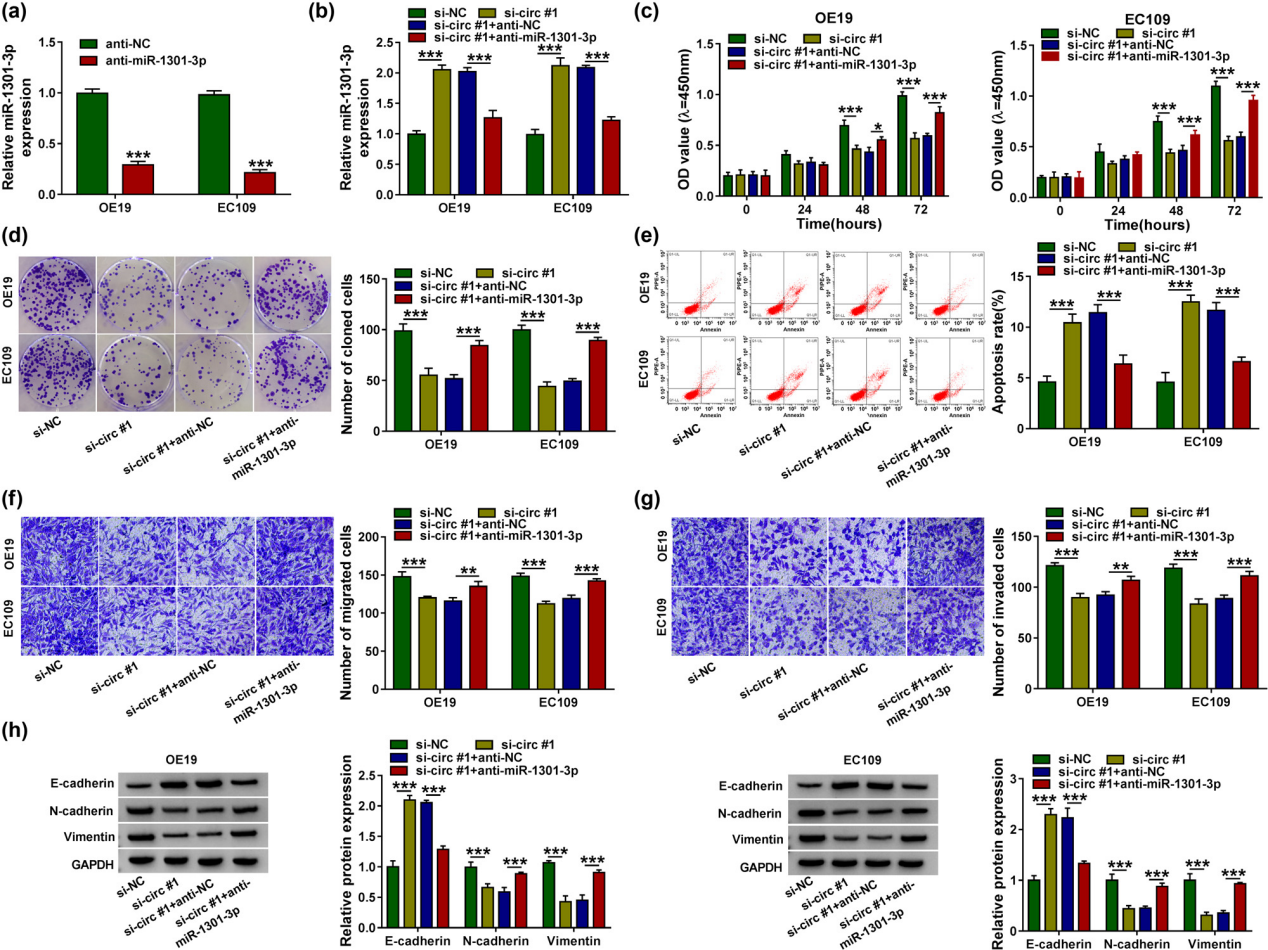
Anti-miR-1301-3p partially rescued the function of circ_0004370 inhibitor. (a) The expression level of miR-1301-3p in cells significantly decreased after inhibition of miR-1301-3p. (b) In OE19 and EC109 cells transfected with si-NC, si-circ #1, si-circ #1 + anti-NC, and si-circ #1 + anti-miR-1301-3p, relative miR-1301-3p expression was detected by RT-qPCR. (c–e) The cell proliferation, cloning, and apoptosis were analyzed by CCK-8, colony formation assay, and flow cytometry. (f and g) The cell migration and invasion were detected by Transwell assay. (h) EMT-related proteins were detected by western blot assay. *P < 0.05, **P < 0.01, ***P < 0.001.

To confirm whether the interaction between miR-1301-3p and circ_0004370 affects the cells function, we first used RT-qPCR to detect the miR-1301-3p expression. As expected, the miR-1301-3p expression was greatly reduced after the transfection of anti-miR-1301-3p (Figure 4a). Interestingly, the expression level of miR-1301-3p was upregulated by circ_0004370 knockdown and restored after the addition of anti-miR-1301-3p (Figure 4b). The cell viability assay showed that the effect of circ_0004370 knockdown was reversed by the miR-1301-3p inhibitor (Figure 4c). The experiment of cell cloning proved that when the expression level of circ_0004370 was downregulated, cell cloning in EC cells was significantly decreased, while the number of cell cloning was recovered after the addition of miR-1301-3p inhibitor (Figure 4d). In cell apoptosis experiments, the number of apoptosis was upregulated by the circ_0004370 knockdown, but it was decreased by addition of anti-miR-1301-3p (Figure 4e). The transwell assay showed that knockdown of circ_0004370 significantly decreased the cell migration and invasion, while inhibition of miR-1301-3p rescued the function of circ_0004370 knockdown (Figure 4f and g). Besides, the western blot analysis showed that the addition of anti-miR-1301-3p rescued the EMT process changes caused by knockdown of circ_0004370 in OE19 cells and EC109 cells (Figure 4h). Taken together, these data determined that miR-1301-3p inhibitor partially rescued the effects of circ_0004370 knockdown on EC cell development.

### 3.5 COL1A1 was a target of miR-1301-3p

**Figure 5:**
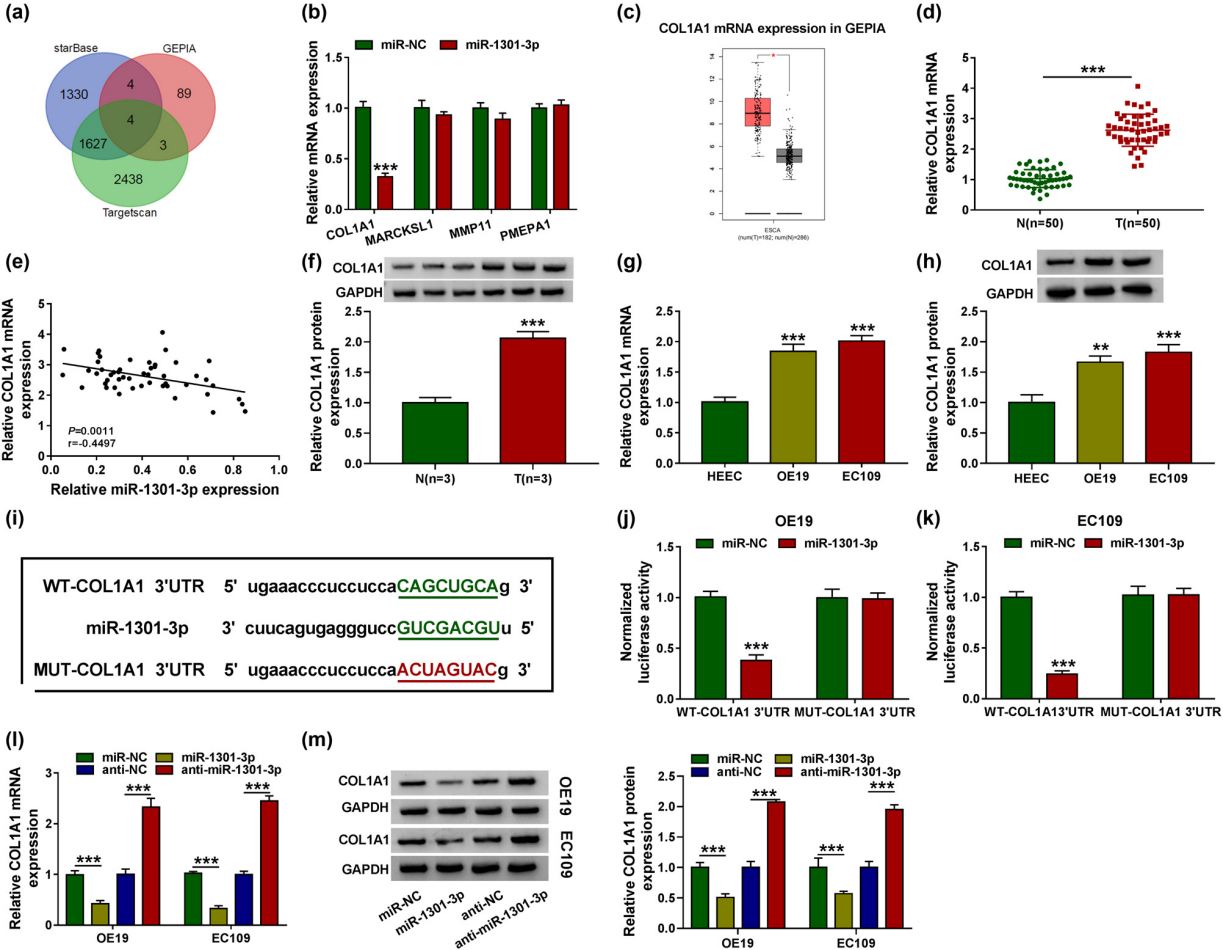
The targeted relationship between miR-1301-3p and COL1A1. (a) Starbase, targetscan, and GEPIA databases were used to predict genes. (b) The mRNA expression level of COL1A1, MARCKSL1, MMP11, and PMEPA1 was detected by RT-qPCR through overexpression of mir-1301-3p. (c) COL1A1 mRNA expression was predicted by GEPIA database. (d) The expression level of COL1A1 was detected by RT-qPCR in EC tissues and adjacent normal tissues. (e) The correlation level between miR-1301-3p and COL1A1 was measured. (f) The COL1A1 expression was detected by western blot analysis. (g and h) The mRNA and protein expression of COL1A1 in EC cells were tested by RT-qPCR and western blot analysis. (i) The binding site of miR-1301-3p and COL1A1 was measured by starBase v2.0. (j and k) The interaction between miR-1301-3p and COL1A1 in OE19 and EC109 cells was determined by dual-luciferase reporter assay. (l and m) The COL1A1 mRNA and protein expression were detected by RT-qPCR and western blot analysis after transfecting with miR-NC, miR-1301-3p, anti-NC, and anti-miR-1301-3p. **P < 0.01, ***P < 0.001.

Previous reports showed that COL1A1 was upregulated and enhanced oncogenicity on EC cells [19]. In order to find the target genes of miR-1301-3p, three independent databases, Starbase, targetscan, and GEPIA, were used to predict genes. Four genes were found in all three databases, namely COL1A1, MARCKSL1, MMP11, and PMEPA1 (Figure 5a). Meanwhile, COL1A1 expression was most significantly reduced among the four genes, so COL1A1 was selected for subsequent experiments (Figure 5b). In the GEPIA database, COL1A1 was highly expressed in EC tissues (Figure 5c). To further verify the COL1A1 expression in EC, we used RT-qPCR to detect the mRNA expression level of COL1A1 and found that the expression level of COL1A1 in EC tissues was increased dramatically (Figure 5d). The expression levels of COL1A1 and miR-1301-3p were negatively correlated in EC tissues (Figure 5e). In western blot assay, the protein expression of COL1A1 in EC tissues was significantly increased compared to the adjacent normal tissues (Figure 5f). Next, RT-qPCR and western blot assay indicated that the expression level of COL1A1 was increased in OE19 and EC109 cells (Figure 5g and h). Besides, results from starBase v2.0 software predicted that there was a binding site between COL1A1 and miR-1301-3p (Figure 5i). In the dual-luciferase reporter experiment, it was further verified that miR-1301-3p could directly bind to 3′UTR of COL1A1 in OE19 and EC109 cells (Figure 5j and k). MiR-1301-3p downregulated the expression of COL1A1 mRNA and protein, and miR-1301-3p inhibitor upregulated the expression of COL1A1 mRNA and protein (Figure 5l and m). Together, it was demonstrated that miR-1301-3p could regulate the expression of COL1A1.

### 3.6 COL1A1 partially rescued the function of miR-1301-3p

**Figure 6:**
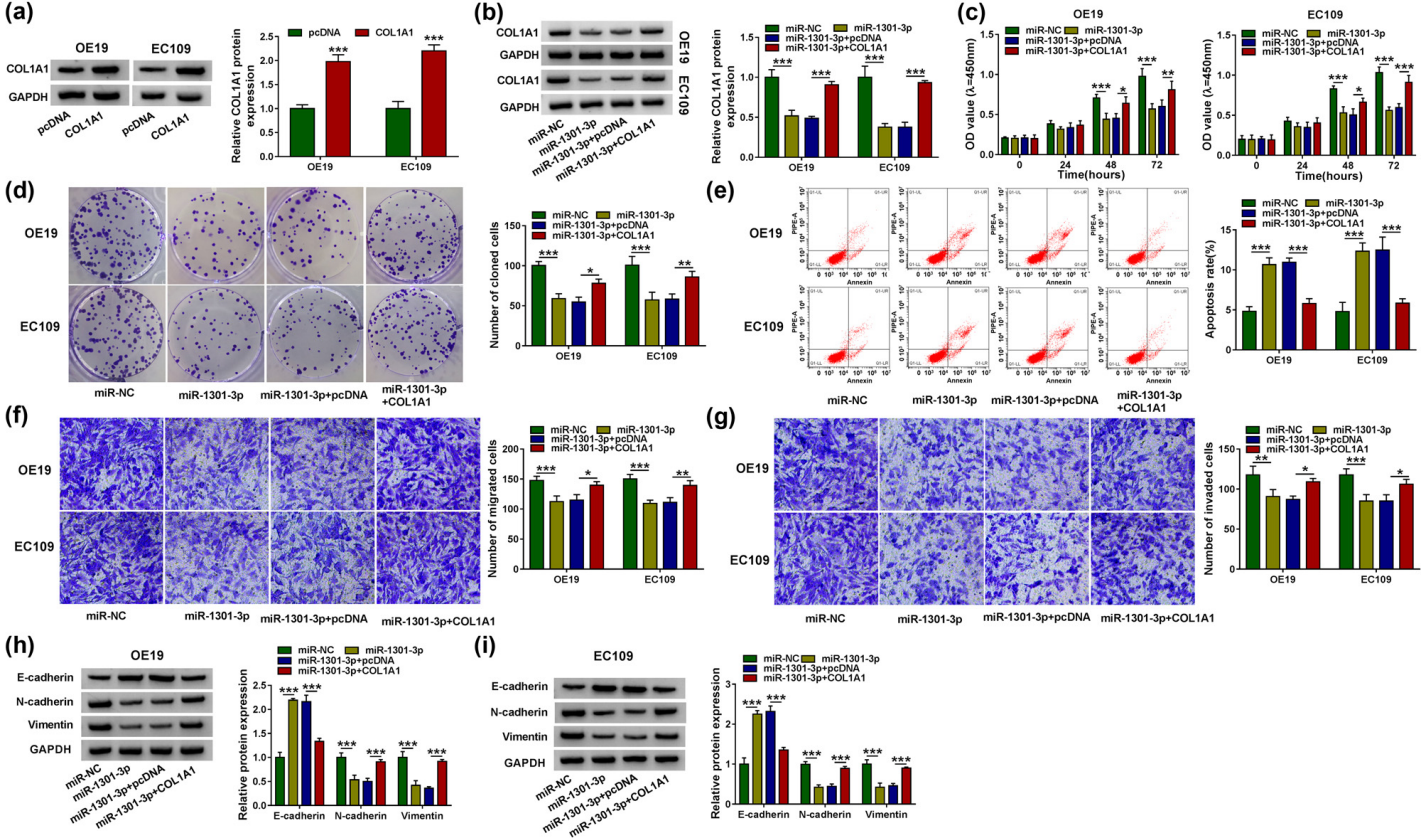
COL1A1 partially rescued the function of miR-1301-3p. (a) The protein expression level of COL1A1 was measured by western blot analysis in OE19 and EC109 cells. (b) OE19 and EC109 cells were transfected with miR-NC, miR-1301-3p, miR-1301-3p + pcDNA, or miR-1301-3p + COL1A1, individually. And relative COL1A1 protein level was detected by western blot analysis. (c–e) The cell proliferation, cloning, and apoptosis were analyzed by CCK-8, colony formation assay, and flow cytometry. (f and g) The cell migration and invasion were measured by Transwell assay after transfecting with miR-NC, miR-1301-3p, miR-1301-3p + pcDNA, or miR-1301-3p + COL1A1. (h and i) EMT marker proteins E-cadherin, N-cadherin, and Vimentin in OE19 and EC109 cells were detected by western blot analysis. *P < 0.05, **P < 0.01, ***P < 0.001.

The western blot analysis was used to measure the COL1A1 protein expression in EC cells. The results showed that COL1A1 protein expression was significantly increased in OE19 and EC109 cells (Figure 6a). Then, we examined the protein expression of COL1A1 after transfecting with miR-NC, miR-1301-3p, miR-1301-3p + pcDNA, or miR-1301-3p + COL1A1. The results showed that the expression level of COL1A1 was decreased after transfecting miR-1301-3p, whereas the protein expression was increased after transfecting with miR-1301-3p + COL1A1 compared with transfection of miR-1301-3p + pcDNA (Figure 6b, *P* < 0.001). We subsequently tested the cell viability using CCK-8 assay. The results revealed that upregulation of miR-1301-3p markedly reduced cell viability in OE19 and EC109 cells, whereas transfection with COL1A1 rescued the cell viability in EC cells (Figure 6c). Then the cell cloning assay proved that cell cloning was significantly reduced in EC cells transfected with miR-1301-3p; however, the number of cell cloning was recovered after transfection of COL1A1 (Figure 6d). The flow cytometry was used to detect apoptosis. The results indicated that transfection of COL1A1 could partially reverse the effects of miR-1301-3p on cell apoptosis (Figure 6e). The results of transwell assay showed that miR-1301-3p significantly decreased the cell migration and invasion, while addition of COL1A1 rescued the function of miR-1301-3p in EC cells (Figure 6f and g). The western blot assay showed that addition of COL1A1 rescued the effect of miR-1301-3p on EMT process in OE19 cells and EC109 cells (Figure 6h and i). We concluded that COL1A1 protein could reverse the effect of miR-1301-3p on EC cells.

### 3.7 Knockdown of circ_0004370 inhibited EC growth *in vivo* via miR-1301-3p/COL1A1 axis

**Figure 7:**
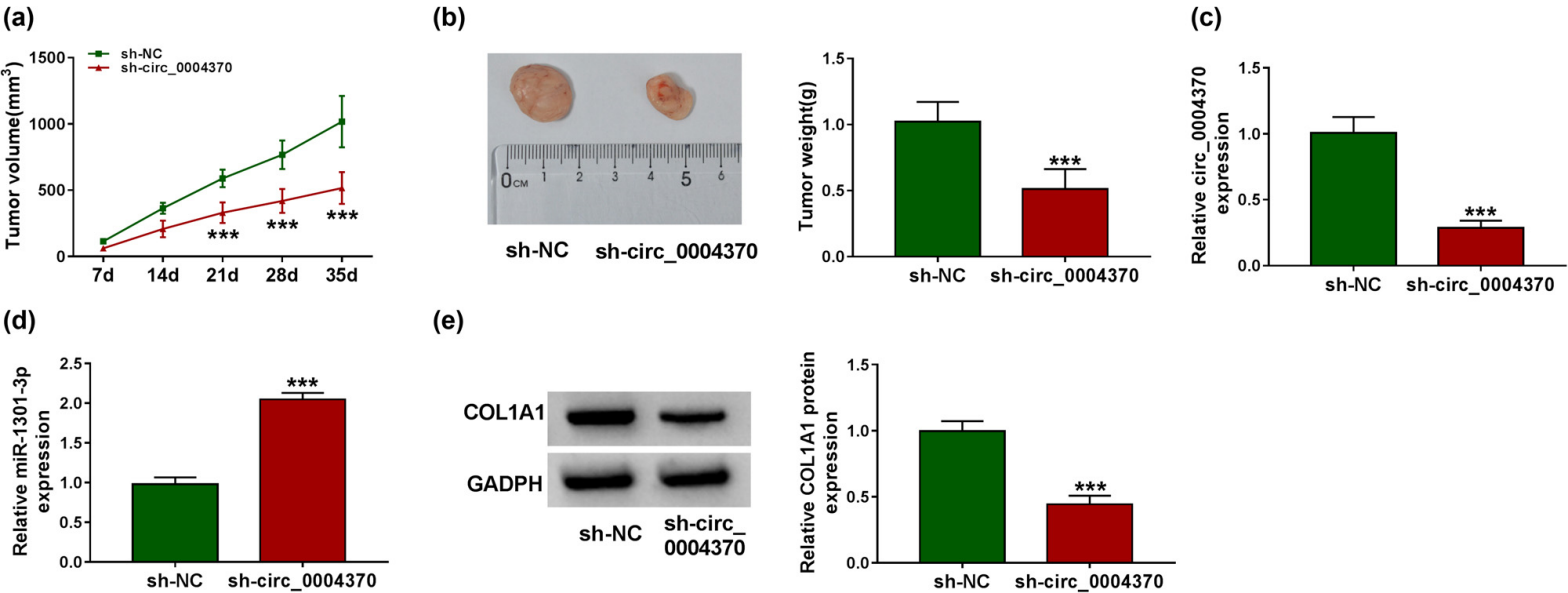
Silencing of circ_0004370 reduced tumor volumes and weights *in vivo.* (a) The tumor volume in the tumor xenograft model after transfecting with the sh-circ_0004370 was measured. (b) The tumor weight in the tumor xenograft model after transfecting with the sh-circ_0004370 was measured. (c) The expression level of circ_0004370 was detected by RT-qPCR when EC tissues were transfected by sh-NC or sh-circ_0004370. (d) The expression level of miR-1301-3p was measured after transfecting with the sh-circ_0004370. (e) The COL1A1 protein expression level was detected by western blot analysis in sh-circ_0004370 group and sh-NC group. ***P < 0.001.

We wondered whether downregulated circ_0004370 reduced the EC tumor growth. We used a xenograft nude mouse model and found that knockdown of circ_0004370 broadly suppressed the tumor volumes and weights (Figure 7a and b). Moreover, the RT-qPCR showed that circ_0004370 was significantly decreased and miR-1301-3p expression was remarkably increased with downregulation of circ_0004370 (Figure 7c and d). Knockdown of circ_0004370 also decreased COL1A1 protein level in tissues (Figure 7e). In conclusion, circ_0004370 promoted EC growth by regulating miR-1301-3p/COL1A1 axis.

## 4 Discussion

EC is a type of high mortality serious tumors worldwide. This is a malignant lesion caused by an abnormality of esophageal squamous epithelial cells or adenocytes. Recently, a growing number of studies have confirmed that noncoding RNAs participate in the pathological process for various cancer [21,22,23]. Circular RNA (circRNA) is a novel noncoding RNA that attracts concerns. CircRNAs are stable structures composed of precursor mRNA back-splicing. At present, many circRNAs have been identified as tumor promoters or inhibitors, which have aberrant expression levels in bladder cancer [24], papillary thyroid cancer [25], oral cancer [26], and colorectal cancer [27]. However, the function of circRNA and the underlying regulatory mechanism remain unclear. In the current research, we concluded that circ_0004370 served as a tumor promoter to activate cell viability, cloning, migration and invasion, and EMT process and restrain cell apoptosis via miR-1301-3p/COL1A1 axis.

Consistent with a previous report which reported the abnormal circ_0004370 expression in EC [8], our study first detected that circ_0004370 was upregulated in EC tissues and cells (OE19, TE11, KYSE410, and EC109). Downregulation of circ_0004370 notably affected EC cellular activities such as cell viability, cloning, apoptosis, migration and invasion, and EMT process. Furthermore, knockdown of circ_0004370 *in vivo* showed that tumor volumes and weights were significantly decreased in xenograft mouse model.

CircRNAs take part in biological processes through multiple regulatory mechanism. Specifically, circRNAs are identified as competitive endogenous RNAs (ceRNAs) to sponge miRNAs, thereby attenuating the inhibitory effects of miRNAs and promoting the expression of miRNAs target genes [28]. Alternatively, circRNAs have the role of regulating gene transcription [29]. In addition, the function of circRNAs is interacting with RNA-binding proteins (RBPs) [30]. We speculated that circ_0004370 might be a ceRNA, with the function of sponging miR-1301-3p. To verify this speculation, we first detected that in the cytoplasmic distribution results, circ_0004370 was highly expressed in cytoplasm. Besides, we predicted that circ_0004370 bound to the miR-1301-3p using online software. Further dual-luciferase reporter analyzed the direct targeting relationship between circ_0004370 and miR-1301-3p. Previous study reported the negative correlation between circTNFRSF21 and miR-1227 in endometrial carcinoma tissues and cells [31]. Consistent with this, in this study we found the markedly inverse correlation between circ_0004370 and miR-1301-3p in EC. Knockdown of circ_0004370 could affect the functions of EC cells, such as cell proliferation, cell cloning, migration, and invasion, whereas miR-1301-3p inhibitor rescued the functions of circ_0004370 knockdown. Our results provided a fresh evidence for the role of circ_0004370 in EC to downregulate miR-1301-3p.

COL1A1 as a type of group I collagen plays a significant role in the development of multiple cancers [32]. The previous study indicated that COL1A1 was associated with the gastric cancer and promoted cell migration and metastasis [33]. Additionally, COL1A1 knockdown suppressed the metastasis of breast cancer cells [19]. In addition, Yin et al. demonstrated that COL1A1 played a crucial role in EC [20]. Recently, with the attention paid to COL1A1, a large number of evidence showed that miRNA had a targeting relationship with COL1A1. For example, it was reported that miR-129-5p inhibited cell viability of gastric cancer by downregulating COL1A1 [34]. In our findings, starbase software predicted that miR-1301-3p directly targeted COL1A1, and further experiments proved that there was a negative correlation between them. Moreover, cell proliferation, apoptosis, migration, and other experiments demonstrated that COL1A1 could restore the effect of miR-1301-3p. Finally, we found that knockdown of circ_0004370 upregulated miR-1301-3p and further downregulated COL1A1 expression. Previous studies exhibited that circNEK6 promoted thyroid cancer progression through Wnt signaling pathway [35]. In addition, circ_100290 played the critical role in colorectal cancer initiation via Wnt/β-catenin signaling pathway [36]. However, it is not clear whether circ_0004370 affects the development of EC by the Wnt signaling pathway, which will be the focus of our future study.

In this study, there are some limitations for *in vivo* experiments. We have detected the effect of circ_0004370 depletion on tumor growth *in vivo*. However, the effects of circ_0004370/miR-1301 3p/COL1A1 axis on tumor metastasis cannot be done currently due to the laboratory conditions. Future works are expected to refine this mechanism in EC using mouse model.

## 5 Conclusions

In conclusion, we discovered circ_0004370 was upregulated in EC cells and tissues. Moreover, as a tumor promoter in EC, circ_0004370 could greatly promote the cell viability, cloning, migration, and invasion, remarkably suppressed apoptosis, and affected EMT process of EC through regulation of miR-1301-3p/COL1A1 axis. Therefore, our study suggested that circ_0004370/miR-1301-3p/COL1A1 axis might be potential therapeutic target in future, which may provide novel direction for the further clinical trials.

## References

[1] Bray F, Ferlay J, Soerjomataram I, Siegel RL, Torre LA, Jemal A. Global cancer statistics 2018: GLOBOCAN estimates of incidence and mortality worldwide for 36 cancers in 185 countries. CA Cancer J Clin. 2018;68(6):394–424. 10.3322/caac.21492 30207593

[2] Pennathur A, Gibson MK, Jobe BA, Luketich JD. Oesophageal carcinoma. Lancet. 2013;381(9864):400–12. 10.1016/S0140-6736(12)60643-6 23374478

[3] Codipilly DC, Qin Y, Dawsey SM, Kisiel J, Topazian M, Ahlquist D, et al. Screening for esophageal squamous cell carcinoma: recent advances. Gastrointest Endosc. 2018;88(3):413–26. 10.1016/j.gie.2018.04.2352 PMC7493990 29709526

[4] Ning S, Li X. Non-coding RNA resources. Adv Exp Med Biol. 2018;1094:1–7. 10.1007/978-981-13-0719-5_1 30191482

[5] Zhang J, Liu H, Hou L, Wang G, Zhang R, Huang Y, et al. Circular RNA_LARP4 inhibits cell proliferation and invasion of gastric cancer by sponging miR-424-5p and regulating LATS1 expression. Mol Cancer. 2017;16(1):151. 10.1186/s12943-017-0719-3 PMC5594516 28893265

[6] Li XN, Wang ZJ, Ye CX, Zhao BC, Huang XX, Yang L. Circular RNA circVAPA is up-regulated and exerts oncogenic properties by sponging miR-101 in colorectal cancer. Biomed Pharmacother. 2019;112:108611. 10.1016/j.biopha.2019.108611 30797148

[7] Sun H, Tang W, Rong D, Jin H, Fu K, Zhang W, et al. Hsa_circ_0000520, a potential new circular RNA biomarker, is involved in gastric carcinoma. Cancer Biomark. 2018;21(2):299–306. 10.3233/CBM-170379 PMC13078270 29103021

[8] Zhang Z, Lin W, Gao L, Chen K, Yang C, Zhuang L, et al. Hsa_circ_0004370 promotes esophageal cancer progression through miR-1294/LASP1 pathway. Biosci Rep. 2019;39(5):BSR20182377. 10.1042/BSR20182377 PMC6522713 30988074

[9] Bartel DP. MicroRNAs: Target recognition and regulatory functions. Cell. 2009;136(2):215–33. 10.1016/j.cell.2009.01.002 PMC3794896 19167326

[10] Backes C, Meese E, Keller A. Specific miRNA disease biomarkers in blood, serum and plasma: Challenges and prospects. Mol Diagn Ther. 2016;20(6):509–18. 10.1007/s40291-016-0221-4 27378479

[11] Zhi T, Jiang K, Zhang C, Xu X, Wu W, Nie E, et al. MicroRNA-1301 inhibits proliferation of human glioma cells by directly targeting N-Ras. Am J Cancer Res. 2017;7(4):982–98. PMC5411805 28469970

[12] Wang L, Zhao Y, Xu M, Zhou F, Yan J. Serum miR-1301-3p, miR-335-5p, miR-28-5p, and their target B7-H3 may serve as novel biomarkers for colorectal cancer. J Buon. 2019;24(3):1120–7. 31424670

[13] Peng X, Yan B, Shen Y. MiR-1301-3p inhibits human breast cancer cell proliferation by regulating cell cycle progression and apoptosis through directly targeting ICT1. Breast Cancer. 2018;25(6):742–52. 10.1007/s12282-018-0881-5 29951881

[14] Zhang C, Xie L, Fu Y, Yang J, Cui Y. lncRNA MIAT promotes esophageal squamous cell carcinoma progression by regulating miR-1301-3p/INCENP axis and interacting with SOX2. J Cell Physiol. 2020;235(11):7933–44. 10.1002/jcp.29448 31943174

[15] Marini JC, Forlino A, Cabral WA, Barnes AM, San Antonio JD, Milgrom S, et al. Consortium for osteogenesis imperfecta mutations in the helical domain of type I collagen: regions rich in lethal mutations align with collagen binding sites for integrins and proteoglycans. Hum Mutat. 2007;28(3):209–21. 10.1002/humu.20429 PMC4144349 17078022

[16] Müller WEG, Ackermann M, Neufurth M, Tolba E, Wang S, Feng Q, et al. A novel biomimetic approach to repair enamel cracks/carious damages and to reseal dentinal tubules by amorphous polyphosphate. Polymers. 2017;9(4):120. 10.3390/polym9040120 PMC6432492 30970799

[17] He X, Lee B, Jiang Y. Cell-ECM interactions in tumor invasion. Adv Exp Med Biol. 2016;936:73–91. 10.1007/978-3-319-42023-3_4 27739043

[18] Liu S, Liao G, Li G. Regulatory effects of COL1A1 on apoptosis induced by radiation in cervical cancer cells. Cancer Cell Int. 2017;17:73. 10.1186/s12935-017-0443-5 PMC5534093 28775672

[19] Liu J, Shen JX, Wu HT, Li XL, Wen XF, Du CW, et al. Collagen 1A1 (COL1A1) promotes metastasis of breast cancer and is a potential therapeutic target. Discov Med. 2018;25(139):211–23. 29906404

[20] Yin Y, Du L, Li X, Zhang X, Gao Y. miR-133a-3p suppresses cell proliferation, migration, and invasion and promotes apoptosis in esophageal squamous cell carcinoma. J Cell Physiol. 2019;234(8):12757–70. 10.1002/jcp.27896 PMC13483280 30537034

[21] Rupaimoole R, Slack FJ. MicroRNA therapeutics: Towards a new era for the management of cancer and other diseases. Nat Rev Drug Discov. 2017;16(3):203–22. 10.1038/nrd.2016.246 28209991

[22] Peng WX, Koirala P, Mo YY. LncRNA-mediated regulation of cell signaling in cancer. Oncogene. 2017;36(41):5661–7. 10.1038/onc.2017.184 PMC6450570 28604750

[23] Zhang HD, Jiang LH, Sun DW, Hou JC, Ji ZL. CircRNA: a novel type of biomarker for cancer. Breast Cancer. 2018;25(1):1–7. 10.1007/s12282-017-0793-9 28721656

[24] Li P, Yang X, Yuan W, Yang C, Zhang X, Han J, et al. CircRNA-Cdr1as exerts anti-oncogenic functions in bladder cancer by sponging microRNA-135a. Cell Physiol Biochem. 2018;46(4):1606–16. 10.1159/000489208 29694981

[25] Bi W, Huang J, Nie C, Liu B, He G, Han J, et al. CircRNA circRNA_102171 promotes papillary thyroid cancer progression through modulating CTNNBIP1-dependent activation of β-catenin pathway. J Exp Clin Cancer Res. 2018;37(1):275. 10.1186/s13046-018-0936-7 PMC6234664 30424816

[26] Chen L, Zhang S, Wu J, Cui J, Zhong L, Zeng L, et al. circRNA_100290 plays a role in oral cancer by functioning as a sponge of the miR-29 family. Oncogene. 2017;36(32):4551–61. 10.1038/onc.2017.89 PMC5558096 28368401

[27] Li XN, Wang ZJ, Ye CX, Zhao BC, Li ZL, Yang Y. RNA sequencing reveals the expression profiles of circRNA and indicates that circDDX17 acts as a tumor suppressor in colorectal cancer. J Exp Clin Cancer Res. 2018;37(1):325. 10.1186/s13046-018-1006-x PMC6307166 30591054

[28] Cui M, Shen W, Qin W, Wang X, Li Y, Xu F, et al. Circular RNA ciRS-7 promotes tube formation in microvascular endothelial cells through downregulation of miR-26a-5p. J Biochem Mol Toxicol. 2020;34(5):e22468. 10.1002/jbt.22468 32053286

[29] Chen LL. The biogenesis and emerging roles of circular RNAs. Nat Rev Mol Cell Biol. 2016;17(4):205–11. 10.1038/nrm.2015.32 26908011

[30] Holdt LM, Stahringer A, Sass K, Pichler G, Kulak NA, Wilfert W, et al. Circular non-coding RNA ANRIL modulates ribosomal RNA maturation and atherosclerosis in humans. Nat Commun. 2016;7:12429. 10.1038/ncomms12429 PMC4992165 27539542

[31] Liu Y, Chang Y, Cai Y. circTNFRSF21, a newly identified circular RNA promotes endometrial carcinoma pathogenesis through regulating miR-1227-MAPK13/ATF2 axis. Aging. 2020;12:6774–992. 10.18632/aging.103037 PMC7202486 32299063

[32] Li J, Ding Y, Li A. Identification of COL1A1 and COL1A2 as candidate prognostic factors in gastric cancer. World J Surg Oncol. 2016;14(1):297. 10.1186/s12957-016-1056-5 PMC5126984 27894325

[33] Wang F, Xue Q, Xu D, Jiang Y, Tang C, Liu X. Identifying the hub gene in gastric cancer by bioinformatics analysis and in vitro experiments. Cell Cycle. 2020;19:1326–37. 10.1080/15384101.2020.1749789 PMC7469508 32293980

[34] Wang Q, Yu J. MiR-129-5p suppresses gastric cancer cell invasion and proliferation by inhibiting COL1A1. Biochem Cell Biol. 2018;96(1):19–25. 10.1139/bcb-2016-0254 28482162

[35] Chen F, Feng Z, Zhu J, Liu P, Yang C, Huang R, et al. Emerging roles of circRNA_NEK6 targeting miR-370-3p in the proliferation and invasion of thyroid cancer via Wnt signaling pathway. Cancer Biol Ther. 2018;19(12):1139–52. 10.1080/15384047.2018.1480888 PMC6301817 30207869

[36] Fang G, Ye BL, Hu BR, Ruan XJ, Shi YX. CircRNA_100290 promotes colorectal cancer progression through miR-516b-induced downregulation of FZD4 expression and Wnt/β-catenin signaling. Biochem Biophys Res Commun. 2018;504(1):184–9. 10.1016/j.bbrc.2018.08.152 30173892

